# Round the Hospitals

**Published:** 1918-09-28

**Authors:** 


### ROUND THE HOSPITALS.

At the Investiture held on September 18 the King conferred the Royal Red Cross, First Class, on the following:—*Q.A.R.N.N.S.*—Superintending Sister Margaret Goodall-Copestake. *Q.A.R.N.N.S.(R.).*—Sister Muriel Hutton. *R.N. Auxiliary Hospitals.*—Matron Ellen Finnemore. *T.F.N.S.*—Matron Katherine Merriman. The following were admitted Associates of the Royal Red Cross:—*Q.A.I.M.N.S.*—Sister Sarah McClelland. *Q.A.R.N.N.S.(R.).*—Sisters Vera Spark and Zoë Stronge. *R.N.A. Hospitals.*—Matrons Margaret Tod, Elsie Philp, and Elizabeth Ritchie. *Q.A.I.M.N.S.(R.).*—Matron Melita Martin, Sisters Henrietta Daly, Edith Davies, Annie Ellis, Ann Gibb, Florence Hale, Lizzie

ROUND THE HOSPITALS—(*continued*).

Haxell, and Maud Reynolds-Knight. *T.F.N.S.*—Sister Charlotte Fitzpatrick. *V.A.D.*—Miss Bessie Ernest and Miss Selina Lloyd. *Canadian A.N.S.*—Sisters Mary Blott and Mildred Parkins.

It will be remembered that Miss E. M. McCarthy, R.R.C., Matron-in-Chief in France, was gazetted in June last a Commander of the Order of the British Empire. This appointment has now been cancelled, and in lieu of it Miss McCarthy has been appointed to be a Dame Grand Cross of the Order. Dame McCarthy's promotion is extremely popular in the Service, with which she has always had the most cordial relations. She is personally known to a very large proportion of the nurses who have served under her in France, and to her is very largely due the admirable nursing organisation and the absence of friction under totally untried conditions. She has easily earned the title of the Queen of Organisers.

The committee of the Royal Victoria Infirmary, Newcastle-on-Tyne, have approved the following rates of pay to the sisters and nurses: Sisters, £55, rising by £5 to £70; first staff nurse (theatre), £45; second staff nurse (instruments), £43; other staff nurses, £40. Probationers: first year, £17 10s.; second year, £21 10s.; third year, £27 10s. This new rate of pay compares very favourably with the former scale, and, indeed, with that of other institutions of the same class.

The Blackburn and East Lancashire Royal Infirmary has not quite reached the level attained at Newcastle. We learn that the board of management has this month adopted a new scale of salaries which marks an advance. The assistant matron is to receive £70; the out-patient and *x*-ray sister, £65; the night sister, £60; the operating theatre sister, £60. Ward sisters, whose salary was formerly £40 rising by a yearly increment of £2 10s. to £45, from now on start at £45 and rise by £5 yearly to £55. Staff nurses, formerly receiving £30 and £36, will have £35 and £40. Probationers' salaries have risen from £10, £18, £20 to £16, £22, £25 per annum.

At the Royal Infirmary, Huddersfield, the salary of probationers has been raised from £8, £12, £16 to £12, £16, and £20. The sisters have received an augmentation during the war raising their salary from £40, £45 to £45, £50. They now have a bonus, in addition, of £5.

A protracted discussion took place at the meeting of the Brighton Guardians on September 17 with regard to the nurses' salaries and the bestowal of a war bonus. Probationers at present receive £15 to £20, staff nurses £28 to £32, ward sisters £35 to £50, superintendent nurse £100. There are forty-six nurses on the staff, and up to the present no war bonus has been given. Considerable opposition was shown to the recommendations of the special committee appointed to go into the question of war bonuses to all the officials, but, so far as the nurses were concerned, all ended happily. An amendment raising the proposed bonus of £5 to one of £7 10s. was passed by eleven to five. The zeal displayed by some of the Guardians in the cause of the nurses was an interesting feature of the debate.

The Queen of the Belgians, who has received a generous gift of a million francs from the American Red Cross, proposes to expend part of it on the household expenses of a large rest-home which she is providing for the nurses of the Belgian ambulances and hospitals. These nurses have shown great devotion since the war broke out, having often tended the wounded in the Belgian Army far up into the front lines. Many have been wounded, and many more are in need of rest and change. The villa Henri IV., at Cannes, has been remodelled, and is being fitted up for their use under the name of the Royal Elizabeth Club. The situation is ideal for the purpose, and the nurses who exchange the shell-pitted and over-moist regions where they have so long been stationed for the sunny Côte d'Azur will have drawn a good lot.

An officer of the Royal Air Force, who had assuredly not been without much vivid personal experience of danger, has been describing the unimaginable horror of an attack for two hours and a half by high explosives and incendiary bombs on the hospital where he lay helpless. He relates that the first bomb completely smashed up four wards like a pack of cards; the second smashed up two or three more; the third set fire to a ward; the fourth fell two wards away and smashed another. "Imagine lying there in bed, sick, hearing that bomb whistling as it came down, not knowing where it was likely to fall, perfectly helpless, and with no cover!" During the whole time the V.A.D. night nurse behaved magnificently, walking up and down the ward with a word of cheer for every patient, making them hot drinks, and showing not the slightest sign of fear.

The raids at Etaples were the more appalling, because the patients were in large proportion men with broken thighs or other injuries which made it advisable they should be spared the journey home. Many of these men, with legs shattered for the second time, are now making a good recovery at a well-known South Coast hospital, in which a number of huts are devoted entirely to leg cases. It has been found that long-continued rest at the outset, with the proper appliances and specialised treatment, can rescue very badly injured men from becoming cripples later on, and a great deal of attention is being bestowed on this branch of surgery, with the most encouraging results. One cheerful youth whom we saw had both thighs broken, not to mention twelve other wounds, but the sister assured us he would be able to walk in good time.

**ROUND THE HOSPITALS**—*(continued)*.

An Army Order recently issued states that the conditions governing the award of the Silver War Badge to the disabled have been amended. The badge will be issued to officers and men who have served with the Colours "for at least seven days subsequent to August 4, 1914," and to various classes of persons in addition who fulfil the same conditions, including "female nurses and members of Voluntary Aid Detachments and Queen Mary's Army Auxiliary Corps, who have been discharged or relinquished their duties on account of physical disabilities such as would render them permanently unfit for further service in their respective corps." It is known that a considerable number of nurses in all the Services are eligible for this badge.

The long-expected has actually happened, and has proved fully as disastrous as was feared. There has been a strike of hospital workers, cooks, and cleaners at the Holborn Military Hospital, Mitcham, containing about 900 patients, and these wounded men were exposed to delayed meals and general discomfort in consequence. It is hard to imagine what their feelings must be at finding themselves without proper attendance after the long-drawn-out agonies of war are over and they are restored to a grateful country. The Holborn Board of Guardians is responsible for this disgraceful dislocation of the hospital service. Fortunately the strike is now ended, the workers having been granted 5s. extra pay weekly, with concessions in regard to hours. The women had long been dissatisfied with their rate of pay, and apparently the authorities took no trouble to make it understood among the workers that their request for an increase would be shortly dealt with. A trifling dispute about the arrangement of the work brought the long-smouldering discontent to a head. The incapacity of the Board in allowing matters to come to this point has been as clearly demonstrated as the want of patriotic feeling in the Mitcham cooks and ward-maids.

The wastage of labour in small auxiliary hospitals is clearly demonstrated by the remarkably efficient kitchen huts at Netley. To feed over 1,000 persons the chief cook has under her but two men and eleven kitchenmaids. Her assistant in a supplementary kitchen hut has two more maids. The kitchen apparatus is a model of scientific adaptation of means to an end, and is packed into so small a space that without personal inspection of the spotless order prevailing it must have been deemed incredible. To feed a similar number of men in small establishments from 60 to 100 persons must have been kept hard at work.

If voluntary hospitals as a rule succeed brilliantly with their children's wards the same cannot be said of the out-patient departments, where in all which deal with children's complaints waiting crowds of little children may be witnessed on the appointed days. There is little doubt that out-patient attendance has a markedly devitalising effect upon the young, and this for two reasons. It confers a kind of demoralising distinction upon the child thus selected for exemption from school, and a long, idle time spent under circumstances calculated to deepen his sense of morbid satisfaction in his complaint. Secondly, in the sensitive and imitative child it weakens resistance to disease. He coughs because he hears consumptive children cough, or instinctively walks crooked because his gaze has been fascinated by a sufferer with a weak spine. All who understand children are aware of the existence of these traits. Out-patients' departments and treatment in adult wards foster them both. They lie at the root of that puzzling modern manifestation "the hospital habit," the foundations of which are often laid in childhood. In a well-organised school clinic the evil is minimised.

A writer in the *Spectator* pleads for the reorganisation of the V.A.D. Nursing Service under the title of a "Woman's Auxiliary Nursing Service." He is under the belief that the existing shortage of women for general service in hospitals is due to their lack of status "as servants of the King." The fact is that in most well-managed auxiliary hospitals vacancies seldom occur, and it is reckoned a favour to get taken in. The work depends far more than any of the other war Services on personal considerations. The girls who undertake it are for the most part still under parental control, and their guardians desire to be assured that they will be considerately treated. The recruiting has to be done individually rather than collectively, and usually matrons and commandants have to do it for themselves, since many of the best candidates recoil from registering at Devonshire House as a kind of leap in the dark. Reorganisation has an attractive sound, but *plus ça change plus c'est la même chose*.

The new Midwives (Ireland) Act has been long awaited. It provides for the establishment of a Central Midwives Board to regulate training courses and examinations for certificates, to publish the roll of certified midwives annually, and to make rules as to discipline, suspension, and cancelling of certificates. The four women members of the Board, appointed by the Local Government Board for Ireland to act as "midwives' representatives," are Miss J. H. Kelly, matron, Maternity Hospital, Belfast; Miss M. Blunden, late matron, Lying-in Hospital, Cork; Miss A. Michie, Superintendent for Ireland, Q.V.J.I.N.; Miss G. O'Carroll, matron, Coombe Lying-in Hospital, Dublin. The Act comes into force on January 1 next, after which all women practising as midwives in Ireland (unless under a doctor) will have to be certified. The lying-in hospitals in Ireland have long had a high reputation, and there is no lack of first-rate teaching. The problem before the administrators of the new Act is to get down to the peasantry and tone up the large body of *bona-fide* midwives at present doing as they please.

☛ For "A Question of Attitude," by "Ierne," see p. 558; Some London Hospital Correspondents, see p. 562.

